# Analgesic efficacy of intrathecal fentanyl during the period of highest analgesic demand after cesarean section: A randomized controlled study: Erratum

**DOI:** 10.1097/01.md.0000496599.67747.13

**Published:** 2016-09-09

In the article “Analgesic efficacy of intrathecal fentanyl during the period of highest analgesic demand after cesarean section: A randomized controlled study”,^1^ which appeared in Volume 95, Issue 24 of *Medicine*, Dr. Bieryło's and Dr. Kołacz's names contained typographical errors. The article has since been corrected online.
